# Analysis of the Actions and Motivations of a Community during the 2017 Torrential Rains in Northern Kyushu, Japan

**DOI:** 10.3390/ijerph17072424

**Published:** 2020-04-02

**Authors:** Atsuko Nonomura, Kazuhito Fujisawa, Mari Takahashi, Hideo Matsumoto, Shuichi Hasegawa

**Affiliations:** 1 Faculty of Engineering and Design, Kagawa University, Takamatsu 7610396, Japan; hasegawa@eng.kagawa-u.ac.jp; 2 Institute of Education, Research and Regional Cooperation for Crisis Management Shikoku, Kagawa University, Takamatsu 7608521, Japan; fujisawa@cc.kagawa-u.ac.jp (K.F.); dcmkikikanri2@jim.ao.kagawa-u.ac.jp (M.T.); h-matsu@cc.kagawa-u.ac.jp (H.M.)

**Keywords:** flooding, evacuation, community, communication, local landforms

## Abstract

Damage caused by weather events has increased dramatically across the world in recent years. In the case of Japan, record-breaking rainfall has caused devastating damage almost every year since 2014; many people have been killed in these disasters. To better prepare for future heavy rainfalls, we need to discover how to prepare for disasters and mitigate damage by learning from examples in resilient communities. In 2017, torrential rains hit Toho Village in northern Kyushu, and the people as a whole responded well to avoid disastrous outcomes. We studied the actions and motivations of residents of Toho during this rainfall event by conducting semi-structured interviews in November 2017. The interviewees indicated that their motivation for evacuating was “personal observation of the danger” or “communication with neighbors”. Communication within the community was found to be an important factor that enabled the safe evacuation of community members, even without notice of the disaster risk and/or in the absence of timely information from the government because of a power outage. Knowledge of local landforms would be also helpful to reinforce appropriate actions and precautions needed during a disaster.

## 1. Introduction

Over the last two decades, damage caused by weather events has increased dramatically and ubiquitously throughout the world [1,2]. The Asia-Pacific region experiences a wide range of natural hazards due to its geographical location and has been particularly disaster prone [3]. Many people living in the region are among the most sensitive and susceptible to extreme weather events and a changing climate. Extreme weather events, such as heat waves and floods, are likely to become even more severe and more frequent with climate warming, and people need to deal with increasingly hazardous environments [4].

Traditionally, the process of managing or mitigating hazards has been based on technical capacities and expertise. This “top-down” approach was developed by government organizations. Under this approach, responsibility for disasters rests almost exclusively with the local government, and local residents are perceived as passive receivers of disaster risk management measures. As a consequence, disaster information provision has tended to be a one-way process for transferring knowledge and information from experts to residents. The approach assumes that the residents can trust the judgement of the government and will follow their advice closely. Without appropriate and timely information for decision making, a community may not be able to avoid devastating damage. However, since natural hazard from rainfall-induced disasters, such as floods, landslides and debris flows, depends on geographic locations and local physical features, it can be difficult for a local government to provide emergency information to each community in a timely manner.

In the last two decades, the importance of self-help (Jijyo in Japanese) and mutual-help (Kyojyo in Japanese) has been understood and spread in Japan. This is the “bottom-up approach”. Responsibilities and initiatives need to be well-balanced between local residents and the government [5]. As an international strategy, the Sendai Framework for Disaster Risk Reduction promotes resilience building using a top-down approach at the global level and disaster preparedness and early warning built up through a bottom-up approach at the community level [6,7,8]. The Sendai Framework sets four specific priorities for action: (1) understanding disaster risk; (2) strengthening disaster risk governance to manage disaster risk; (3) investing in disaster risk reduction for resilience; and (4) enhancing disaster preparedness for effective response.

## 2. Resilience in Hazard Researches

The concept of “resilience” has also been used at several levels, including the city, community and individual [9,10]. Researchers have analyzed several aspects of resilience and related activities in many case studies [11,12,13,14,15]. Tiernan et al. [10] shows the number of published papers with both words of disaster and resilience in their titles per year since 2000. The number shows a rapid increase since 2009.

The definition of “resilience” is different at several levels and several perspectives. [9,10,16]. Norris et al. [17] provides a comprehensive review of the resilience definitions, which are applied to several levels, such as ecological system, city, social, community and individual. In hazards approach, the definition of resilience is the ability to survive and cope with a disaster with minimum impact and damage. It incorporates the capacity to reduce or avoid losses, contain the effects of disasters and recover with minimum disruptions [16]. Resilience is generally focused on pre-event measures to prevent hazard-related damage (e.g., Bruneau et al. [18]) or post-event strategies to cope with and minimize disaster impact (e.g., Paton et al. [19]). Researches on preparedness, response and vulnerability reduction are related to resilience of pre-event measures (e.g., Paton and Johnston [20]).

According to research interests, the word “resilience” is used as an outcome or a process [16]. Outcome-related resilience is defined in terms of the ability to bounce back or cope with a hazard event. Process-related resilience is defined in terms of the ability to make better decisions and improve capacity to handle hazards.

In our research, we analyzed the response and action by focusing on pre-event and process-related resilience in community level.

## 3. The Aim of This Study

In the case of Japan, record-breaking rainfall has caused devastating damage almost every year since 2014; for example, the 2014 Hiroshima landslide [21], the 2015 Kinu River flood [22], the 2017 July Northern Kyushu torrential rainfall [23] and the heavy rainfall of July 2018 [24]. In addition, many people were killed by these rainfall disasters. To prepare for future heavy rainfalls, we need to analyze the disaster to make it clear why in some areas the number of causalities is small even there may have been many causalities in the disaster. If there are some areas that had few to no causalities, we need to investigate how the people responded to the rainfall and what they did during the rainfall. Learning from the experiences of resilient communities should be a good lesson for us for disaster prevention measures.

In 2017, torrential rains fell in Toho Village in northern Kyushu. Unfortunately, three people died due to debris flow [25], but many people were able to save their own lives. It may be considered that effective measures were taken in various places. For example, it is reported that in a school, Toho Gakuen, the teachers decided that all the students and teachers should stay at school until things settled down in order to protect parents from being affected while they went to and from the school to pick up their children [26]. They stayed one or two nights at school, and their parents were not in danger for picking their children. Toho Village had torrential rains in 2012 as well, and based on their lessons from the 2012 torrential rainfall, the teachers also responded properly to the 2017 torrential rains. In this research, we infer that there were also appropriate responses in the community to save their own lives. The aim of this study is to make clear what was going on, and what they did in the community during the torrential rains. By learning lessons from their response in community, appropriate responses and motivation of the responses were identified. The basic questions are: “How did they avoid disaster?” and, “What was the motivation of their responses?”

## 4. Study Area

**Figure 1:**
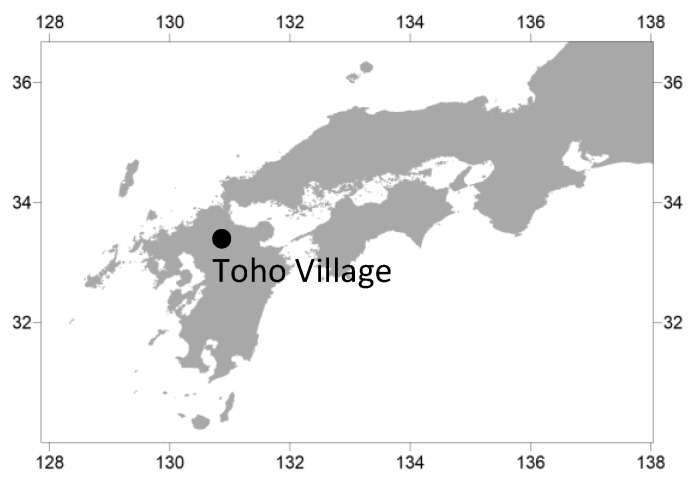
Location of the study area.

**Figure 2:**
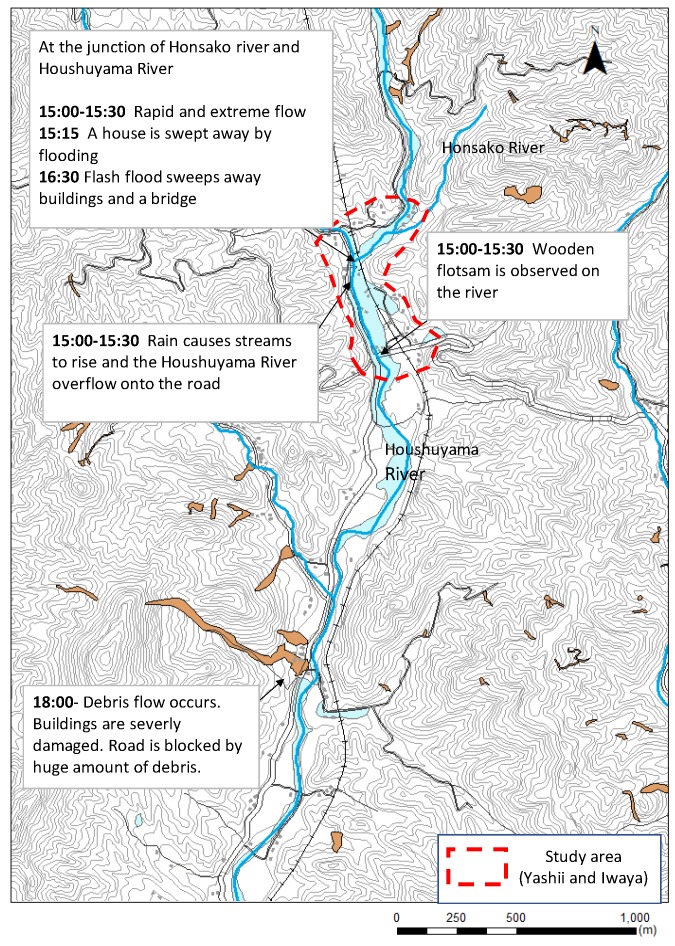
Rivers in the study area and a timeline (Japan Standard Time) of flooding on 5 July 2017. The study area is outlined in red.

Toho Village is located in Fukuoka Prefecture in northern Kyushu (33°26′ N, 130°53′ E) (Figure 1). Area is 51.97 km^2^. Elevations in the area range from 150 to 700 m, with the higher elevations in the north. The number of people is 2135 and the number of households is 871 in 2018 [27]. The study area consists of residential areas located on gently sloping debris-flow fans and river terraces along the Houshuyama River and its tributary, the Honsako River (Figure 2).

On 5 July 2017, torrential rains in northern Kyushu caused floods, debris flows and landslides. The Japan Meteorological Agency urged “utmost vigilance” in much of Fukuoka Prefecture, saying the heavy rain could bring about a “once in 50-year” disaster. Fukuoka Prefecture had approximately 600–1000 mm of rain in a 24-h period, and many houses were destroyed or damaged by debris flows and flooding [28].

**Figure 3:**
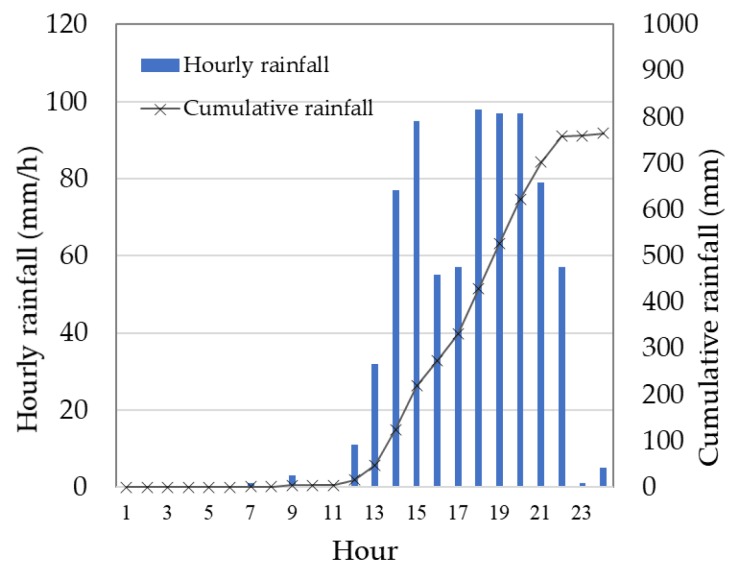
Hourly rainfall observed at the Toho Village Office on 5 July 2017.

**Figure 4:**
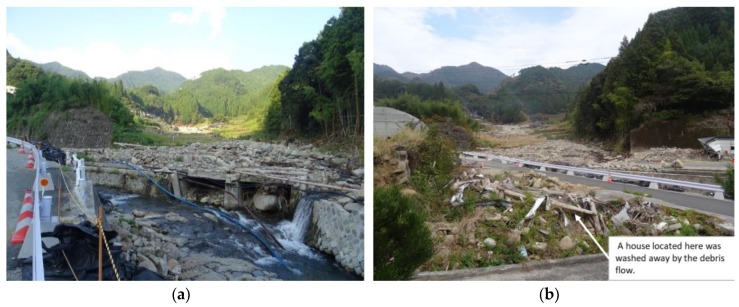
Photos of the Yashii area: (**a**) debris flow damage along the Honsako River and (**b**) the remains of a house that was washed away near the junction of the Honsako and Houshuyama Rivers.

Toho Village had 765 mm of rain in a 24-h period on 5 July 2017 (Figure 3). The maximum of 30-years average July daily rainfall is 17.9 mm/day at the nearest Automated Meteorological Data Acquisition System, Asakura, [29]. The rain in a 24-h period on 5 July 2017 is more than 40 times the average year value. At 13:14 on 5 July, a heavy rain flood warning was issued and an alert was broadcasted at 13:30. In response to the real-time landslide risk warning at 14:40, evacuation preparation information was issued at 14:17. Moreover, an evacuation advisory was issued at 15:15 [30] (Table 1). Debris flows swept down the Houshuyama and Honsako Rivers, and some houses and a bridge were swept away. In the Yashii and Iwaya areas, the damage was especially severe, and three people were killed [25] (Figure 4a,b).

Toho Village had torrential rains in 2012 as well. Although some people said that experiences of the 2012 rainfall helped their decision in this rain, they were more seriously damaged by the 2017 rainfall. Many of them said that they had never experienced such a severe rainfall damage.

## 5. Methods

Investigations were carried out in two areas where especially severe damage was caused by flooding and debris flow, Yashii and Iwaya. A semi-structured questionnaire was used during face-to-face interviews. By visiting all the households in the areas, we intended to interview 1 person per household. In the areas, 46 households and 198 people were registered. Since some houses were absent during the survey, 23 households were interviewed. Interviews were carried out by the members of Kagawa University’s science team and staff of Toho Village during 6–9 November.

Each interview took about 30 min and all of the interviews were audio-recorded. During the interview, the following three questions were asked:

Question 1. Where were you, what happened around you and what did you do during the 2017 torrential rains in northern Kyushu? What influenced your actions during the heavy rain?
Question 2. What triggered (motivated) your actions? How did you evacuate? (This question was only for people who evacuated.)
Question 3. Do you take any daily basis, safety precautions to protect yourself from natural disasters caused by the river or/and mountain? If people notice the signs of debris flow or/and flooding—and find differences from usual rain as early as possible— they can properly response to the hazards in advance. Therefore, we asked this question about daily basis safety precautions against mountains and rivers, which lead to appropriate response to avoid damage.

## 6. Results

### 6.1. Overview of the 2017 Torrential Rains in Northern Kyushu

**Figure 5:**
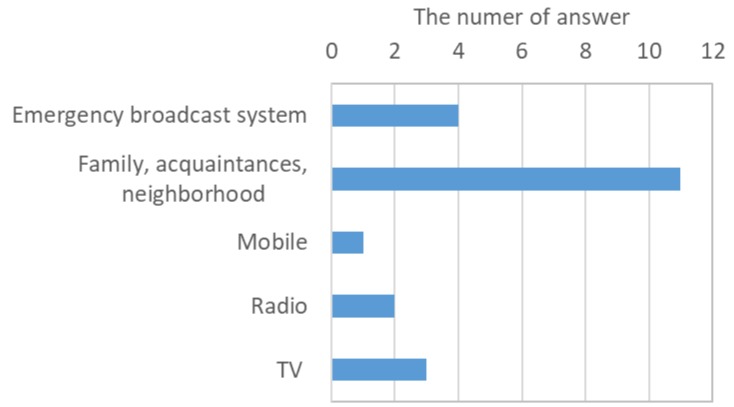
Information during the 2017 torrential rainfall in the study area.

In the afternoon of 5 July, heavy rain was observed at the office of Toho Village (Figure 3). At 14:17, an evacuation advisory was issued by the local government. The respondents identified events that occurred in the study area and actions taken by the residents. By combining their accounts of events, we were able to create the sequence shown in Figure 2. Around 14:00, some parts of the roads along the river began to be submerged. Around 15:00, the rain caused the streams to rise and the Houshuyama River overflowed onto the nearby road and a house was swept away by floodwater along the Honsako River. At the same time, a widespread power outage occurred, and landline telephone service was lost. At 16:30, a debris flow occurred in the Honsako River and some buildings and a bridge were washed away (Figure 4a). Although alerts were issued through emergency broadcast system, radio and TV, respondents obtained alert information mainly from their family, acquaintances and neighborhoods (Figure 5). The main information for them was obtained from family, acquaintances and neighborhood.

### 6.2. Activity during the Flood (Questions 1 and 2)

**Figure 6:**
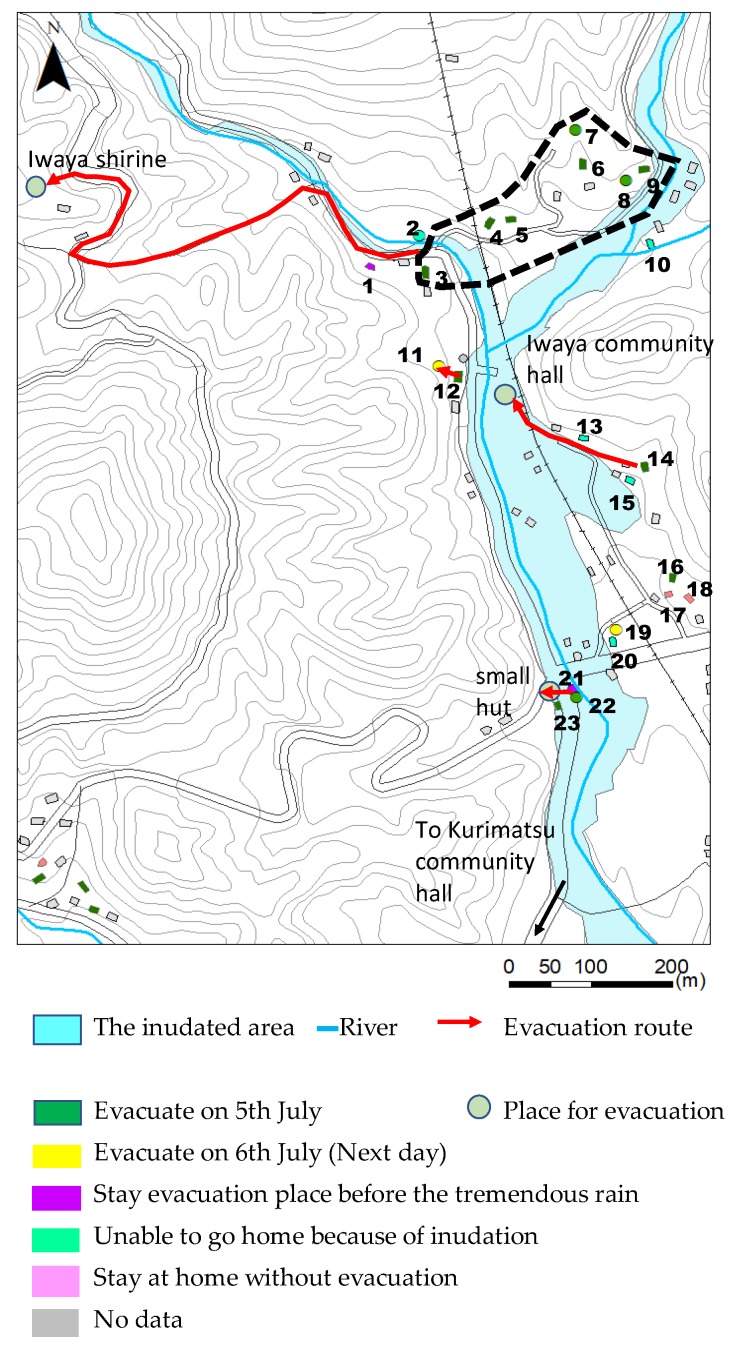
Actions taken in the study area.

**Figure 7:**
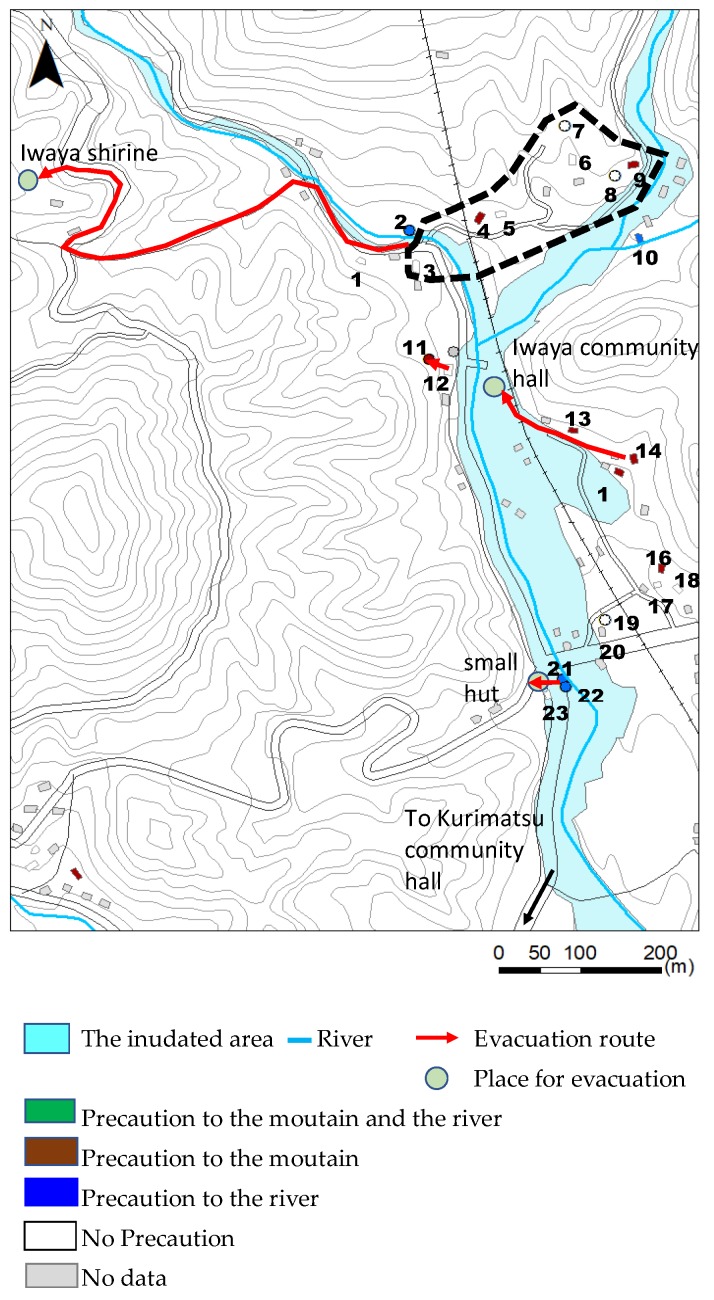
Precautions taken in the study area.

Data gathered about residents’ activities and motivations during the rain event (questions 1 and 2) are summarized in Table 2. The locations of the ID numbers shown in Table 2 correspond with the positions of the numbers shown in Figure 6 and Figure 7. Figure 6 shows the evacuation activities of each family based on the interview results. Houses, of which residents evacuated on 5 July are colored green. Houses, of which residents evacuated on 6 July, after tremendous rain, are colored yellow. Houses, of which residents stayed at an evacuation place before the tremendous rain are colored purple. Some elderly respondents were at a public recreational facility as usual. During their stay, since it began to rain heavily, they decided to stay there until things settle down. The public recreational facility was functionalized as an evacuation place. Houses, of which residents could not go home or stayed away from home, are colored light green. They could not go home because roads were closed due to inundation and debris flows. Houses, of which residents stayed at home, are colored pink. They considered that going out and standing outside were dangerous in the tremendous rain.

#### 6.2.1. Yashii

On 5 July, most of the people in the Yashii area (households 1–12) either evacuated their homes or were unable to return home from their workplace because of road inundation. Around 15:00, several extraordinary sights began to be observed; these include extremely high flow, rising river water levels, a debris flow and a washed away building. After witnessing a flash flood and a house washed away by the debris flow, household No. 5 reached out to nearby community members about evacuating around 15:00. Households 4–9 held a discussion at the house of No. 5.

They discussed the followings. As compared to 2012 rainfall, the rain seems much stronger. A part of the house of No. 5 was already washed away. It was too dangerous to stay here. Although their designated evacuation place was the Iwaya Community Hall, it seemed dangerous for them because it was located near the junction of Honsako River and Houshuyama River. They decided to evacuate to a building at the Iwaya shrine, which was located on higher ground across the river, together. At about 15:30, they traveled by car to the shrine. After their evacuation, around 16:30, a debris flow occurred, and some buildings and a bridge were washed away along the Honsako River (Figure 4a). These interviewees said that their motivation for evacuating was “talking with neighbors” or “phone calls from their families, neighbors or relatives” who suggested evacuation. The early and voluntary evacuation most likely saved at least some of their lives. There was a case that early and voluntarily evacuation saved lives of people in Kamaishi [31].

Respondent No. 12 witnessed the debris flow from their house at 16:30, and a house located next to that of No. 12 was swept away by the debris flow (Figure 4b). She worried that the next debris flow might hit her house and evacuated to the house of respondent No. 11, staying one night with her children. The house of No. 12 was highly exposed to the risk of debris flow. Her personal observation of the danger and ability to communicate with her neighbor were an effective way to avoid the danger posed by the disaster. Since the road in front of their houses was flooded by fast moving water like a river, they decided to stay at the house of No.11. Next day, when police patrol officer came to the house of No.11, they realized the surrounding area was seriously damaged by flooding and debris flows. Then, they decided to evacuate into the building at the Iwaya shrine.

#### 6.2.2. Iwaya

Respondents No. 13–No. 23 were from the Iwaya area. Two households (No. 14–No. 15) evacuated to the Iwaya Community Hall, which was a designated evacuation location (Figure 6). Because the road was already inundated by the time that they left their homes, it took an hour for them to reach the Iwaya Community Hall, which was only about 200 m from their houses. Three other respondents (No. 17–No. 19) stayed in their own houses because they thought that staying home was safer than evacuating to the Iwaya Community Hall. Another respondent (No. 16) evacuated to a different community hall (Kurimatsu Community Hall), which was about 500 m farther than the nearest hall (Iwaya Community Hall) after an evacuation preparation information was issued by the local government at 14:17. The Iwaya Community Hall is located on a terrace about 20 m from the Houshuyama River. During a frequent heavy rain, the community hall is available as an evacuation location. However, under a tremendous rain such as the one on 5 July, it was not an appropriate place for evacuation because the area surrounding the building is inundated.

**Figure 8:**
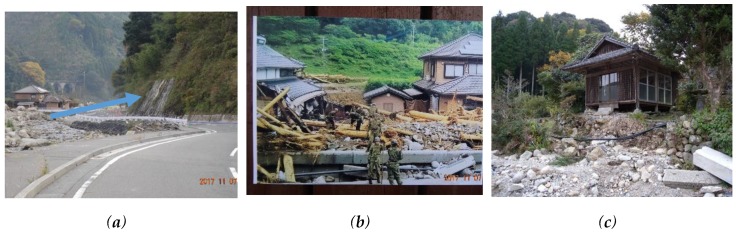
Photos of the Iwaya area: (**a**) an inundated road, a blue arrow indicates the direction of flooding during the rain, (**b**) the house of the respondent No. 22, which was damaged by flooding and (**c**) a small hut exposed to the flooding.

Respondents from households No. 22 and No. 23 evacuated to a hut located on slightly higher ground because the road near their houses had already started to be inundated and they could not go to the Iwaya Community Hall (Figure 8a). Since these houses were located next to the river, they were exposed to flood risk (Figure 8b), but the hut they went to was also vulnerable to flooding (Figure 8c).

### 6.3. Daily Basis Safety Precautions to Protect Yourself from Natural Disasters Caused by the River or/and Mountain (Question 3)

During the rain event, the power failed at about 15:00 and television could not be used as a source of information provision in some areas. Many of the residents had to make decisions about what to do in the absence of timely information about the ongoing disaster. In the absence of timely information about the ongoing disaster, their own decision affects life and death. Daily basis safety precautions were one of the most important factors to make the right decision and protect their own lives. During the interview, respondent No. 11 said that she and her husband were concerned about a mountain stream flowing behind their house during the rain, and the amount of water increased than usual. Respondent No. 22—who lives along the Houshuyama River—said that she and her husband were concerned about the water level of the river because the water level rose faster than before since the riverbed was raised by the runoff debris after the 2012 tremendous rain. During the rain, they watched the water level and noticed the situation was unusual. If people notice the signs of debris flow or/and flooding as early as possible, they can properly respond to the hazards in advance by themselves. If a person is concerned about a mountain stream flowing behind his/her house or the water level of a nearby river and has daily basis safety precautions against mountains and rivers, the person might notice the abnormal phenomenon early. It might make possible to respond early to the rainfall-induced hazards. Therefore, we asked this question about daily basis safety precautions which lead to appropriate response to avoid damage.

During the interview, we asked the respondents about their daily basis safety precautions to protect themselves from natural disasters caused by the river or mountain (Figure 7). In the Yashii area, only a few respondents take daily basis safety precautions to protect themselves from natural disasters. However, most of the respondents evacuated to a higher place and saved their lives. They decided to evacuate through a neighborhood discussion. In this area, there is a good neighborhood relationship in the community. The case of Yashii demonstrated that the combination of a few people’s daily basis precautions and the neighborhood relationship protect the community. The good neighborhood relationship is also one of the important factors for a resilient community, namely, to protect their own lives from disaster.

In the Iwaya area, along the Hoshuyama River and Yashii River, flood hazard maps are not made. In Japan, along rivers that may cause serious damage to the national economy, flood hazard maps are made. However, along middle and small rivers, flood hazard maps are not made. In order to know where is the flood-hazardous area, substitute information is necessary. Along the Hoshuyama River, terraces are distributed. In a frequent heavy rain, there is no flood risk on the upper terrace. However, in a heavy rain, flood risk needs to be considered because the upper terrace was also formed by previous tremendous flooding. In the Iwaya area, some people who lived at the foot of the mountains on the upper terrace take daily basis safety precautions only against the mountain but not against the river. If people take daily basis safety precautions against the river on the upper terrace, all the people will be able to choose the appropriate places for evacuation and avoid the danger. The knowledge of the landform can support people to understand the hazardous area of flooding caused by tremendous rainfall.

## 7. Discussion

In hazards research, the word “resilience” means the ability to survive and cope with a disaster with minimum impact. In the case of noticed events such as typhoon, hurricane and cyclone, the course and strength are forecast and early warning information is widely provided. During Cyclone Sidr in 2007, successful evacuation of coastal residents was reported [32], although there are still problems that not everyone evacuated, even they received a cyclone warning [33]. In the case of tsunami triggered by earthquakes generated in a subduction zone, although there is no forecasting information about earthquake occurrence beforehand, it is well known that evacuation to the higher places is necessary [31]. In those natural hazards, in order to evacuate to safe zones, bonding social capital, which is the close relationships between individuals, is effective for dealing with the hazards [34,35].

On the other hand, in the case of record breaking heavy frontal rain event, since it is not a noticed event, people are forced to decide if they need to evacuate or not during the rainfall. It is difficult to issue evacuation orders for the local government to the community because the risk of sediment related disaster and flooding depend on the local topography. In 2009, Sayo Town, there was a record-breaking heavy frontal rain event during 8–11 August 2009. During the rainfall, there were eight casualties who were only trying to evacuate early to an evacuation center near their residences before the local government issued an evacuation advisory. After the accident at 21:20, the local government issued an evacuation advisory to the whole area of the town based on the reports of inundation provided by residents. However, more than half of the people did not evacuate to the designated evacuation places and stayed at their houses because they considered that evacuation was more dangerous than staying at home under heavy rainfall in the nighttime [36]. During the frontal rain, people should decide their actions by themselves. Therefore, if people living in unprotected floodplains and coastal locations and if the people were unfamiliar with local hazards and ways of coping with them, the record-breaking heavy frontal rain event may cause serious damage. Since the frequency of record-breaking heavy rainfall increases recently, the cases which people responded well and avoided disastrous outcomes need to be analyzed to identify factors that led to the successful evacuations.

In Toho Village, while an evacuation advisory was issued, no evacuation order was issued because the Mayer decided that for people, especially for elderly people to go out under tremendous rainfall was very dangerous. Under such situation, people decided what to do by themselves.

In the Yashii area, only a few people took daily basis safety precautions to surrounding mountain and river. Although some people noticed that the rain was extraordinary, the discussion they had was a key factor for them to decide evacuation. Most of the respondents evacuated to a higher place and saved their lives. In this area, there is a good neighborhood relationship in the community. The case of Yashii demonstrated that the combination of people’s precautions, awareness and the neighborhood relationship protect the community. The good neighborhood relationship is also one of the important factors for a resilient community, namely, to protect their own lives from natural disasters.

In Iwaya area, the surrounding of the designated evacuation place, Iwaya Community Hall, was inundated. The Iwaya Community Hall is located on an upper terrace. In a frequent heavy rain, there is no flood risk on the upper terrace. However, in a heavy rain, flood risk needs to be considered. There were several different cases in people’s action: some people went to the designated evacuation place of this area, some people stayed at home and some people evacuated to other places, which were not their designated evacuation place. People were forced to make their own decisions without enough information. In Japan, along the middle and small-scale rivers, hazard maps are not made. Along the Hoshuyama River and Yashii River, flood hazard maps are not made. On the other hand, hazard maps of debris related hazard was created in Toho Village [37]. As a result, the debris related damage areas are designated but flooding hazard area was not designated. This may mislead and confuse people to understand about the hazardous areas. Since the Iwaya Community Hall is located outside of the debris related hazard area, it seems safe during rainfall on the hazard map. It was difficult for people to decide their action under the tremendous rain, which they had never experienced before.

Along the Hoshuyama River, terraces are distributed and the Iwaya Community Hall is located on the upper terrace. In a frequent heavy rain, there is no flood risk on the upper terrace. However, in a heavy rain, flood risk needs to be considered because the upper terrace was also formed by previous tremendous flooding. Some people who lived at the foot of the mountains on the upper terrace take daily basis safety precautions only against the mountain and not against the river (Figure 7). If people take safety precautions against the river on the upper terrace, all the people will be able to choose the appropriate places for evacuation. Greater knowledge of local landforms would be helpful for residents to reinforce capacity to appropriately deal with infrequent disastrous rainfall. To prepare for the next tremendous rainfall, residents need to plan the timing and location of evacuation to a safer place by themselves. The knowledge of the local landform can support people to decide evacuation places to save their own life.

## 8. Conclusions

We examined the case of the tremendous rainfall on 5 July 2017 in Toho Village. Two points stand out in terms of mitigating the damage from this type of rainfall disaster.

1) The people in the Yashii area made a decision to evacuate from the disaster, which may have saved their lives. Especially in the absence of immediate information (i.e., in this case, the power was out so the TV and other sources of information were unavailable), all the people were not aware that the rain was extraordinary. However, some people noticed that the rainfall was heavier than 2012 tremendous rain. It urged them to have a discussion. The discussion they had were a key factor for them to decide evacuation. In this area, there is a good neighborhood relationship in the community. The case of Yashii demonstrated that the combination of people’s precautions, awareness and the neighborhood relationship protect the community. The good neighborhood relationship is also one of the important factors for a resilient community, namely, to protect their own lives from natural disasters.
2) Because the budget and effort are limited, flood hazard maps cannot be prepared for all of the rivers. In Japan, flood hazard maps are not made for middle and small rivers. Therefore, it is difficult for people to know the flooding hazards along the middle or small rivers in Japan. On the other hand, knowledge of local landforms would be helpful for residents to reinforce their capacity to appropriately deal with infrequent disastrous rainfall. Since landform interpretation is usually difficult for ordinary local residents, it is necessary for expert to teach landforms, its formation and hazards to the local residents. The risk communication between local residents and experts must strengthen the community resilience.

## Figures and Tables

**Table 1:** Timeline of Toho Village, Fukuoka Prefecture and Fukuoka Regional Headquarter, Japan Meteorological Agency [30].

Time	Action
13:14	▪ **Heavy rain flood warning** was issued by Fukuoka Regional Headquarters, Japan Meteorological Agency (JMA) ▪ Disaster alert headquarter was established at Toho Village
13:30	▪ **An alert** was broadcasted through emergency broadcast system by the disaster alert headquarter of Toho Village
14:10	▪ **Real time landslide risk warning** was issued by Fukuoka Regional Headquarters, JMA ▪ Disaster alert headquarter was established in Fukuoka prefecture
14:17	▪ **Evacuation preparation information** was issued over all the Toho village by Disaster alert headquarter of Toho village
15:15	▪ **Evacuation advisory** was issued over all the Toho village by Disaster alert headquarters of Toho village
15:30	▪ Disaster Management Headquarters of Toho village was established ▪ Disaster Management Headquarters of Fukuoka prefecture was established
17:51	▪ **Emergency warning for heavy rainfall** was issued by Fukuoka Regional Headquarters

**Table 2:** Conditions, actions taken and motivations for those actions during the 2017 flood (results from interview questions 1 and 2).

ID	Neighbor Relationship	Location	Conditions	Actions	Motivation
1	Good	Not home			
2	-	Not home			
3	-	Home		She decided to evacuate after getting information about occurrence of landsides from a neighbor. She evacuated to a higher place across the river with a nephew living nearby by his car.	Getting information about occurrence of landsides from a neighbor.
4	Good	Home	She heard the roar of the river in the afternoon. 15:30: Power outage	She evacuated to a higher place across the river with neighbors by car.	Talking with neighbors
5	Good	Home office	Sudden strong rain occurred in the afternoon 15:15: Saw a house washed away	He decided to evacuate after talking with neighbors. He evacuated to a higher place across the river with neighbors by car.	Witnessing a house be washed away
6	Good	Home	15:00–15:30: Heard the roar of the river and witnessed splashes 15:30: Telephone not working	She decided to evacuate after talking with neighbors. She evacuated to a higher place across the river with neighbors by car.	Talking with neighbors
7	Good	Home	15:00: Heard the roar of the river and witnessed splashes	He decided to evacuate after talking with neighbors. He evacuated to a higher place across the river with neighbors by car.	Talking with neighbors
8	Good	Home	Too afraid to look outside	She decided to evacuate after talking with neighbors. She evacuated to a higher place across the river with neighbors by car.	Talking with neighbors
9	-	Home	Various things were flowing around her home.	She evacuated to a higher place across the river with neighbors by car.	Phone call from her son to evacuate
10	-	Not home			
11	Good	Home	14:30: The road was submerged, and the car was almost soaked. The rain was so heavy. 15:00: Power outage	She stayed at home on 5 July with her husband and neighbors who had evacuated their home. On 6 July, they evacuated to a higher place across the river where other people were.	Police patrol
12	-	Home	15:00: Power outage 15:30: Flooding river 16:30: Debris flow occurred. Some buildings and a bridge were washed away	She evacuated to a neighbor’s house with her children on 5 July and to higher ground on 6 July.	Witnessing the debris flow
13	Good	Not home	He could not go home because of inundation.		
14	Good	Home	She saw the river had overflowed and saw wooden flotsam in the floodwater	She and her husband evacuated to the Iwaya Community Hall, which was designated as an “evacuation place”.	Looking at the river and mountain conditions
15	Good	Not home			
16	Good	Home	She saw a bridge had been damaged and buildings along the river were damaged.	He evacuated to Kurimatsu Community Hall with his wife.	Evacuation preparation information issued by the local government
17	Good	Home	Roads were inundated around the house.	He stayed home.	Thinking that his home was safer than the community hall
18	Good	Home	Roads were inundated around her house and her house was isolated.	She stayed home because roads were inundated around her house and her house was isolated.	
19	Good	Home	The road flooded and a huge rock, wood and a broken steel frame were moving with the floodwater.	She stayed at home on 5 July because she thought that staying home is safer than evacuation	
20	-	Not home			
21	Good	Not home	All family members were not home. They learned of the conditions through neighbors who sent photos.		
22	-	Home	15:00: Road flooded	She evacuated to a nearby hut located on higher ground.	The road in front of their house flooded.
23	Not so good	Home	Because of wooden flotsam, the house was damaged.	He evacuated to a nearby hut located on higher ground.	Because of driftwood, the house was damaged.
